# Using Different Error Handling Strategies to Facilitate Older Users’ Interaction With Chatbots in Learning Information and Communication Technologies

**DOI:** 10.3389/fpsyg.2021.785815

**Published:** 2021-12-08

**Authors:** Weijane Lin, Hong-Chun Chen, Hsiu-Ping Yueh

**Affiliations:** ^1^Department of Library and Information Science, National Taiwan University, Taipei, Taiwan; ^2^Department of Psychology, Department of Bio-Industry Communication and Development, National Taiwan University, Taipei, Taiwan

**Keywords:** human-robot interaction, older users, literacy education, error handling strategies, voice user interfaces, chatbot

## Abstract

To support older users’ accessibility and learning of the prevalent information and communication technologies (ICTs), libraries, as informal learning institutes, are committed to information literacy education activities with friendly interfaces. Chatbots using Voice User Interfaces (VUIs) with natural and intuitive interactions have received growing research and practical attention; however, older users report regular frustrations and problems in using them. To serve as a basis for the subsequent design and development of an automated dialog mechanism in senior-friendly chatbots, a between-subject user experiment was conducted with 30 older adults divided into three groups. The preliminary findings on their interactions with the voice chatbots designed with different error handling strategies were reported. Participants’ behavioral patterns, performances, and the tactics they employed in interacting with the three types of chatbots were analyzed. The results of the study showed that the use of multiple error handling strategies is beneficial for older users to achieve effectiveness and satisfaction in human-robot interactions, and facilitate their attitude toward information technology. This study contributes empirical evidence in the genuine and pragmatic field of gerontechnology and expands upon voice chatbots research by exploring conversation errors in human-robot interactions that could be of further application in designing educational and living gerontechnology.

## Introduction

In response to the popularity of information and communication technologies (ICTs) and the increasing proportion of older adult users, facilitating older users’ access to information is gaining research and practical attention. The public and private sectors are both investing in the research and development of gerontechnology and service design (Subasi et al., 2011; American Library Association, 2017). As important information agencies and social educational institutes, libraries actively use information technologies to provide older patrons with resources, including collections, services, activities, and facilities that meet their psychological, physical, and information needs. In addition to the technical services which mostly involve library automation infrastructure, few yet significant endeavors have involved the adoption of information technologies to offer reader services that involve social interaction, such as library guidance or book finding service robots (Lin et al., 2014; Tatham, 2016), a reading companion robot (Yueh et al., 2020), and a computer skill tutor robot (Waldman, 2014), all of which used Voice User Interfaces (VUIs) to achieve communication with the users. It has been found that novice users prefer VUIs over keyboards, and VUIs are regarded as highly accessible for older users due to the affordance of natural, intuitive, and easy interaction (Portet et al., 2013; Ziman and Walsh, 2018). However, our reception and interpretation of auditory stimuli are limited by innate physiological mechanisms such as linear processing, which reduce overall comprehension (Yankelovich, 1996), and by acquired psychological factors such as low self-efficacy, which reduce willingness of interaction (Bulyko et al., 2005). These mechanisms and factors make misinterpretation and errors inevitable. Previous studies of human communication with humans and artificial beings suggest that, when errors occur, both parties will attempt to repair the conversation, and the strategies they adopt to handle errors will also affect the users’ conversation behaviors and performance (Clark and Brennan, 1991; Oulasvirta et al., 2006). Despite sporadic discussions on the quantitative and qualitative nature and effectiveness of error-handling strategies, including single vs. multiple uses (Portet et al., 2013; Opfermann and Pitsch, 2017) and re-prompt vs. suggestion styles (Oviatt et al., 1998; Bulyko et al., 2005), the findings have been inconsistent and taken little account of the specific user characteristics of older adults. Previous studies has focused on developing dialog system using alternative error handling strategies other than reprompt for general user models, therefore lacking a comprehensive comparison of all error handling strategies in real use contexts. Furthermore, since the user characteristics are highly associated with the technological affordance of the system interface and modalities, these studies also suggested more research attention on users’ behaviors, performance, and preferences (Oviatt et al., 1998; Bulyko et al., 2005; Portet et al., 2013; Lu et al., 2017). More empirical and systematic investigations on older users’ interactions with VUIs are needed as a basis for designing adaptive voice AIs and voice services. This study therefore aimed to explore how older users interact with a voice chatbot that uses different error-handling strategies. Based on the systematic investigation on older users’ conversational behaviors, performances, and experiences from the error handling perspective, this study intends to present a senior-friendly VUI conversation model as a basis for designing conversational AI chatbots in the future.

In addition to error handling strategies, the motivation and performance of older users in using ICTs are also affected psychologically by their self-efficacy (Bandura, 2006; Hsiao and Tang, 2015). While previous studies of human–computer interaction suggest that older users possess relatively low beliefs and self-perceptions about their ability to use technology to access information and accomplish their goals (Hajiheydari and Ashkani, 2018), studies of human–robot interaction have further indicated that when interacting with more human-like agents, users’ self-efficacy varies due to emotional and cultural influences (Pütten and Bock, 2018). Older users’ low self-efficacy of ICT use is also affected by their physical and cognitive deterioration. As aging decreases the ability to retrieve and recognize words, older users become less sensitive to sounds and take longer to find the right words to express themselves (Baba et al., 2004). In terms of cognitive processing, older adults are less likely to suppress interference from errors or misinterpretation, and they have difficulty recalling old information to associate it with new input (Lee, 2015), both of which create obstacles to their interaction with VUIs. When older users experience difficulties in accessing information due to the abovementioned physical and psychological decline, more frustration, stress, and self-condemnation are reported (Rodin, 1986; Myers et al., 2018), and they are more likely to make errors. As a consequence, it would also prevent them from interaction, learning or the tasks they were to perform. To help older users escape this vicious cycle and have better user experiences, VUIs need not only to receive and recognize user commands but also to help users handle errors in real time and provide adaptive feedback and guidance. Previous studies support that active intervention by a conversational agent can reduce the occurrence of errors (Bulyko et al., 2005), and this study further incorporated different error handling strategies comprehensively in designing voice chatbot guidance and responses to investigate effective error-handling strategies for older users in the real use contexts. In the design of a senior-friendly VUI, the specific research questions to be answered in the present study included (1) Whether there were differences in the behaviors and performance of homogeneous older users when interacting with chatbots with different error-handling strategies? and (2) How the older users with different levels of self-efficacy interacted with the voice chatbots, and whether their performance and preferences differed.

## Materials and Methods

**Figure 1:**
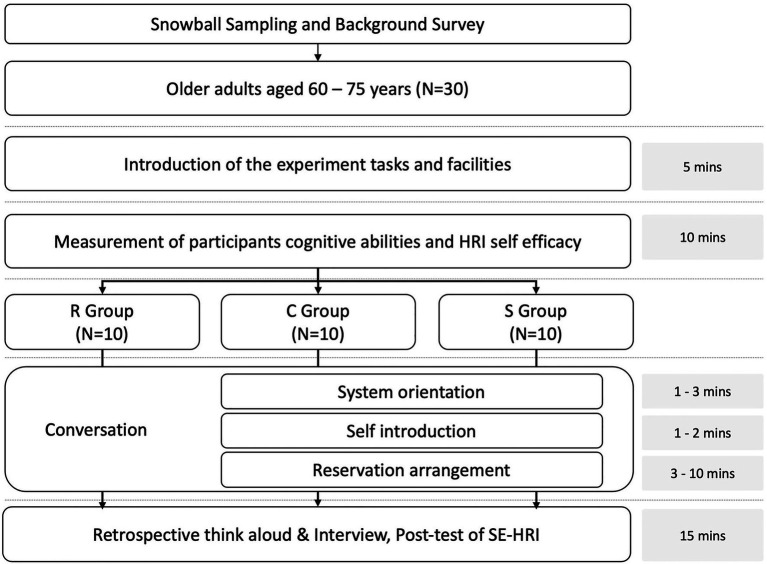
The procedure of the experiment.

The present study adopted the methodology of user experiment with a between-subjects design to investigate how the different error handling strategies of voice chatbots affect older users’ interaction behaviors and performance. The apparatus of the study were three voice chatbots designed with different levels of error handling in terms of the types and amount of system repair initiations. In order to avoid unnecessary technical interferences (Klemmer et al., 2000), the voice chatbots were prototyping through the Wizard-of-Oz technique with the human researchers simulated the speech system. The procedure of the user experiment was illustrated in Figure 1. Within the interactional task of making a restaurant reservation, this study comprised three phases of conversation: system orientation, self-introduction, and reservation arrangement at a restaurant, each with a set of questions and formulaic beginnings of utterances programmed to acquaint older users with the voice chatbots. As shown in Table 1, this study designed and developed three types of voice chatbots, namely *Reprompt (R)*, *Reprompt + Confirm (C)*, and *Repromt + Suggestion (S)* chatbots with reference to different error-handling strategies discussed in previous studies (Oviatt et al., 1998; Bulyko et al., 2005; Bohus and Rudnicky, 2008; Paek and Pieraccini, 2008; Portet et al., 2013; Opfermann and Pitsch, 2017; Ziman and Walsh, 2018).

**Table 1:** Design of the experimental apparatus.

Chatbot	Error-handling strategies in use	System initiation	Examples
R	Reprompt	Repeat the same question when not receiving a correct response	“Have you had lunch yet?”
C	Reprompt + Confirm	Repeat the user response and ask new questions when not receiving a correct response	“It’s not yet noon. Does it mean that you have not yet had lunch?”
S	Reprompt + Suggestion	Inform the user of the system’s expected response, and repeat the same question when not receiving a correct response	“Have you had lunch yet? You can answer whether you have eaten or not.”

This study employed purposeful and non-probability sampling technique of snowball sampling (Parker et al., 2019) to recruit older adults in the fields of public libraries and community centers. A total of 30 older adults aged 60–75 years (*M* = 68.7, *SD* = 4.2) voluntarily participated in the experiment. Their prior knowledge and experiences of ICTs and VUIs were investigated during the recruitment with a self-developed Background Survey. Before the experiment, the participants’ cognitive abilities were measured with the Saint Louis University Mental Status Exam (SLUMS; Tariq et al., 2006), and their self-efficacy in interacting with technologies was measured with the Self-Efficacy in Human-Robot-Interaction Scale (SE-HRI; Pütten and Bock, 2018). According to the pre-tests, all 30 participants had normal cognitive functioning according to the SLUMS criteria. Eighteen (60.0%) of them had previously used the voice assistants of their mobile phones, and 28 (93.3%) had frequently used computers to access and process information.

Participants with different amounts of experience were evenly assigned to the three groups for interaction with the voice chatbot. They heard the prompts using different error handling strategies read by the wizard *via* the voice chatbot with the entire session of the experiment recorded by two video cameras to capture their utterances, facial expressions, and gestures. After the experiment, the retrospective think aloud protocol was used in interview to confirm participants’ behavior intentions. The interview consisted of 14 open questions to investigate participants’ comprehension of the chatbot utterances, perceived efforts, and feelings in the experiment session, and the reasons for their subjective preferences. Also the post-test SE-HRI was distributed to test older adults’ perceived control, confidence, easiness, and satisfaction toward the voice chatbots. The Research Ethics Committee of the University approved all procedures, the protocol, and the methodology (NTU-REC 201907HS018).

## Results and Discussion

Averagely, the participants of all three groups spent nearly 6 min (*M* = 339.8 s) talking to the voice chatbot and completing the restaurant reservation task. The ANOVA was used to compare the performance of the three groups of users, and the results showed that subjects who interacted with Chatbot C, which used reprompt and confirmation error handling strategies, spent significantly more time than did those interacting with Chatbot S and Chatbot R (*F* = 5.7159, *p* < 0.01). As shown in Table 2, the number of back-and-forth alternations between the participants and Chatbot C was the largest. Meanwhile, the participants in Group R were the fastest to complete the task, but they spent a larger percentage of their time dealing with errors, while Group C spent more time in proceeding conversation with the voice chatbot. In addition, participants’ uses of error handling strategies were influenced by the chatbots they encountered. From the interviews, it was found that the participants in Group C regarded their voice chatbot as more human-like, and they were more likely to interact with it in a human-like manner, assimilating their conversational behaviors and actively reducing their uncertainty through additional words. As one participant reflected “*It’s like teaching a child to talk, I could tell the robot what was wrong and expect improvement*” (P29) since she felt the chatbot’s asking questions about everything just like novice and curious children did. Conversely, in Group S, the participants regarded the voice chatbot as a more typical machine; thus, they were more likely to treat it as a subordinate or to express their needs directly and briefly. Participants in Group R had different response strategies depending on their experience or the characteristics of the robot interaction.

**Table 2:** Participants’ conversational performance within three groups.

Group	Strategies	N	Average number of turns	Average completion time (s)	Average number of errors	Average error rate	Satisfaction
R	Reprompt	10	230	297.9	9.40	0.0315	67.2/90
C	Reprompt + Confirm	10	268	328.6	8.50	0.0255	65.5/90
S	Reprompt + Suggestion	10	240	393	9.90	0.0246	64.0/90
Mean (*M*)				339.8	9.27	0.0272	65.6/90

It was also found that when errors occurred in the conversations, compared to the single and common strategy of reprompt, the use of multiple handling strategies, namely, suggestion and confirmation in addition to reprompt, reduced the error rate. According to the triangulation of observation and participants’ satisfaction, albeit less preferred by the older users, the “reprompt + suggestion” strategy was found to be the most effective way to handle conversation errors because the system provided clear guidance to reduce uncertainty and unnecessary trials, resulting in fewer conversational turns and lower error rate. The error handling strategy of “reprompt + confirmation” was less effective due to the lack of clear suggestions, so the participants could more easily become trapped in error loops. However, the analysis of participants’ subjective satisfaction suggested that even with higher error rates, the older users were still able to achieve satisfaction, echoing previous findings in human–robot interaction where the social robot triggered users’ mental models and expectations of human–human interaction (Lohse, 2011). Robots that make mistakes appear more human and thus are easier to accept and trust (Aronson et al., 1966; Salem et al., 2013).

The analysis of the errors revealed that the “reprompt” strategy alone was unable to reduce the number of user errors. This finding is in line with previous studies suggesting that older adults are less experienced and therefore also less aware of the technological affordance of chatbots, so they would guess or generate their responses blindly without identifying what caused the errors (Opfermann and Pitsch, 2017). The fact that older adults spend more time on word finding (Schmitter-Edgecombe et al., 2000) could be associated with the higher error rate in Group R, in which the chatbot used the single error handling strategy of reprompt without providing any guidance for the older adults to repair the conversation.

Further examination of participants’ self-efficacy and conversational performance using paired-sample *t*-test to compare the scores of pre- and post- SE-HRI revealed that the older adults with lower self-efficacy in the beginning perceived more control and capabilities after interacting with the voice chatbots, but those who had higher self-efficacy showed a significant decrease (*t* = 3.33, *p* < 0.05), indicating that high self-efficacy users tended to rely less on the voice chatbots to complete tasks. As shown in Table 3, those with lower self-efficacy in the beginning perceived significantly more control and capabilities in Group S (+5.4) and Group C (+2.6). Those in Groups S and C with higher self-efficacy also experienced decreases, but the decrease was smaller in Group S (−2.8) than in Group C (−8.2). However, despite the participants with lower self-efficacy having the largest increases in self-perception and conversational effectiveness in Group S, they felt the least satisfied and were disappointed with the voice chatbots using the reprompt and suggestion strategies to handle errors. According to the interview results, they expected the voice chatbot to be smarter than a human but found that they could only answer with the limited options suggested. “*I felt a little annoyed that I could only answer the chatbot’s questions with a limited number of choices. I thought it should be smarter*” (P15). Low self-efficacy participants were the most satisfied with the voice chatbot using reprompt and confirmation strategies because they regarded the chatbot as smart in “*pointing out their errors*” (P03) to eliminate uncertainties in the conversation.

**Table 3:** Changes in participants’ self-efficacy.

Group	SE-HRI	*N*	Pre-test Self-efficacy	Post-test Self-efficacy
R		10	65.9/90	58.9/90
	H	5	72.6/90	60.0/90
	L	5	59.2/90	57.8/90
S		10	65.6/90	66.9/90
	H	5	71.2/90	68.4/90
	L	5	60.0/90	65.4/90
C		10	65.5/90	62.7/90
	H	5	72.8/90	64.6/90
	L	5	58.2/90	60.8/90

A noteworthy result from the triangulation of interview and observation data suggested that those participants with high self-efficacy had a rich imagination of the chatbot’s capabilities and tended to keep guessing at the cause of system errors by themselves when such errors occurred, leading to overcorrection and frequent changes in their own responses and error handling strategies. In addition, the more confident they felt in carrying out the conversation with the voice chatbots, the more likely it was that they would give up when they could not complete the task as expected. The “reprompt + suggestion” strategy provided them with enough information to recover from errors and also revealed the limits of the chatbot’s capabilities, thus increasing their confidence in controlling the machine. Therefore, the findings supported that for high self-efficacy older users, the system error handling strategy should still reflect the nature of a machine. On the other hand, low self-efficacy users tended to attribute the errors to their own mistakes, such as inappropriate wording or pronunciation, and were more willing to repeat themselves with minor changes. For them, the “reprompt + confirmation” strategy seemed more effective because they felt encouraged when the voice chatbot, a machine, acted like a human in actively handling the errors along with them.

## Conclusion

This study investigated how different error-handling strategies affected older users’ conversation performances and satisfaction with the voice chatbots. In summary, voice chatbots, which handled errors in conversation provided older users, especially those with lower self-efficacy, with more user control, resulting in higher engagement and performance. According to the comparison of different combinations of error handling strategies, the results suggested that the use of multiple error handling strategies, namely, suggestion and confirmation in addition to reprompt, is beneficial for older users to achieve effectiveness and satisfaction by reducing the error rate and improving their self-efficacy. They were generally more willing to proceed the conversation and explore new tasks including using ICTs under the facilitation of the voice chatbots, which supported the efficiency and importance of error-handling VUIs. Furthermore, the different error handling strategies used by the voice chatbots triggered different expectations of the older users, so that those of similar background and experiences had different ways of responding. The voice chatbot using confirmation to handle errors was regarded by the older users as more human-like, while the one using suggestion was regarded as more machine-like. More importantly, the older users who were less confident in using ICTs had a significant increase in self-efficacy after interacting with the error handling voice chatbots using suggestion and confirmation strategies. And the chatbot using suggestion to handle errors was also preferred by high self-efficacy users because it provided sufficient and efficient guidance for them to complete their tasks.

Based on these preliminary findings, it is suggested that older users’ self-efficacy toward ICTs should be included in the design considerations of VUIs, in general, and instructive VUIs specifically, to provide corresponding mechanisms of error handling. Methodologically, this study contributes to the field studies of gerontechnology by examining comprehensively the different error handling strategies of VUIs in real use contexts with the specific user group of older adults. While several limitations including a one-off session experiment and a relatively small sample size should still be noted, the systematic investigation made by this study on older users’ conversational behaviors, performances, and experiences from the error handling perspective could serve as a basis for designing conversational AI chatbots in the future since it has found critical elements for designing senior-friendly VUIs. The results of this study can further inform the design considerations of any application of chatbots providing information services, including living technology and learning technology in formal and informal education.

## Data Availability Statement

The raw data supporting the conclusions of this article will be made available by the authors, without undue reservation.

## Ethics Statement

The studies involving human participants were reviewed and approved by Research Ethics Committee of National Taiwan University. The patients/participants provided their written informed consent to participate in this study.

## Author Contributions

WL: conceptualization, methodology, visualization, investigation, and writing-original draft. H-CC: software and investigation. H-PY: funding acquisition and writing-review and editing. All authors contributed to the article and approved the submitted version.

## Funding

This study is supported by the Taiwan Ministry of Science and Technology (MOST107-2923-S002-001-MY3 and MOST106-2410-H-002-093-MY2).

## Conflict of Interest

The authors declare that the research was conducted in the absence of any commercial or financial relationships that could be construed as a potential conflict of interest.

## Publisher’s Note

All claims expressed in this article are solely those of the authors and do not necessarily represent those of their affiliated organizations, or those of the publisher, the editors and the reviewers. Any product that may be evaluated in this article, or claim that may be made by its manufacturer, is not guaranteed or endorsed by the publisher.
